# Selection of essential medicines for South Africa - an analysis of in-depth interviews with national essential medicines list committee members

**DOI:** 10.1186/s12913-016-1946-9

**Published:** 2017-01-07

**Authors:** Velisha Ann Perumal-Pillay, Fatima Suleman

**Affiliations:** Discipline of Pharmaceutical Sciences, School of Health Sciences, Westville Campus, University of KwaZulu-Natal, Private Bag X54001, Durban, 4000 South Africa

**Keywords:** Essential medicines, Essential medicines lists, Selection of essential medicines, South Africa, Standard treatment guidelines

## Abstract

**Background:**

The South African (SA) public health system has employed an Essential Medicines List (EML) with Standard Treatment Guidelines (STGs) in the public sector since 1996. To date no studies have reported on the process of selection of essential medicines for SA EMLs and how this may have changed over time. This study reports on the decision making process for the selection of essential medicines for SA EMLs, over the years, as described by various members of the National Essential Medicines List Committee (NEMLC) and their task teams.

**Methods:**

Qualitative in-depth interviews, guided by an interview questionnaire, were conducted with 11 members of the SA NEMLC and their task teams (both past and present members) during the period January – April 2015. Interviews were recorded and transcribed verbatim. Transcripts were then coded by the first author and verified by the second author before being reconciled and input into NVIVO, a qualitative software, to facilitate analysis of the data.

**Results:**

The interviews conducted suggest that the NEMLC process of medicine selection has been refined over the years. This together with the EML review process is now essentially predominantly an evidence based process where quality, safety and efficacy of a medicine is considered first followed by cost considerations which includes pharmacoeconomic evaluations, and pricing of medicines.

**Conclusions:**

This is the first study in SA to report on how decisions are taken to include or exclude medicines on SA national EMLs and provides insight into the SA EML medicine selection, review and monitoring processes over time. The results show that the NEMLC has undergone tremendous transformation over the years. Whilst the membership of the committee largely remains unchanged, the committee has developed its policies and processes over the years. However there is still a need to strengthen the monitoring and evaluation aspects of the SA EML policy process.

## Background

The healthcare landscape in South Africa (SA) has undergone considerable change since the instatement of the country’s new democratic government in 1994 and the subsequent adoption of the National Drug Policy (NDP) for SA, published in 1996 [1]. The country has since been engaging in healthcare reform to ensure equitable access to healthcare and medicines for all citizens, especially those previously disadvantaged by the racially fragmented and under-resourced healthcare services created by the apartheid system [2]. The healthcare system in SA is still a two tier system consisting of both public and private sectors of healthcare. The public sector has three levels of care (PHC, secondary hospital and tertiary/quaternary hospital). One of the key goals of the NDP for SA, through the essential drugs programme (EDP), was to establish a Ministerial appointed National Essential Medicines List Committee (NEMLC) who was responsible for the development of an essential medicines list (EML) for use in the public healthcare sector and to prepare standard treatment guidelines (STG) for the health professionals [1]. This goal was attained when the country’s first STG and EML was published for Primary Healthcare (PHC) in 1996 [3].

Since then there have been 12 editions of the STG/EML published for two levels of healthcare, viz. a PHC book and separate books for adult and paediatric hospital level. The tertiary/quaternary level EML is a list of recommendations and non-recommendations of treatment for specific conditions. This is a constantly updated document and is only available on the National Department of Health (NDoH) website [4]. The SA EML is a list of medicines derived from the STGs.

The NDP explains that one of the drivers for the development of the SA EML was the deficiencies in the pharmaceutical sector such as the rising costs and irrational use of medicines [1]. The creation of an EML was expected to lower the costs of medicines and improve the rational use of medicines by ensuring a limited number of medicines considered essential would be listed and procured [1, 5]. The sector-wide procurement division of the NDoH is responsible for selecting essential medicines, developing STG/EMLs, administration of health tenders, and licensing of individuals and facilities responsible for delivering pharmaceutical services [6]. In a middle-income country setting like SA and in a time of economic crisis and rising costs, there needs to be controls in place to ensure efficient use of the resources for medicines, whilst improving the rational use of medicines, but without compromising the quality of healthcare delivered. The World Health Organization’s (WHO) statement on the advantages of having an EML based on the judicious selection of medicines [7] supports this idea:

“The careful selection of a limited number of essential medicines results in a higher quality of care for patients, better management and use of medicines and more cost-effective use of health resources. Clinical guidelines and lists of essential medicines may improve the availability and proper use of medicines within health care systems. Selection of medicines follows market approval of a pharmaceutical product which defines the availability of a medicine in a country. An essential medicines list may then be developed based on disease prevalence, evidence on efficacy and safety, and comparative cost-effectiveness”.

Thus essential medicines are regarded as medicines that satisfy the priority health care needs of the population. The implementation process of the essential medicines concept must ensure it is flexible and adaptable to suit the needs of the country and selection of essential medicines is a national obligation [8].

The process of selection of medicines in South Africa, in line with STGs, is not easily available in the public domain. Little is known about the NEMLC processes for medicine selection and how these have changed over time. There also seems to be a lack of published information on suggestions and ways to improve the SA EML policy implementation as it evolves over the years, which could benefit other low and middle income countries. This study aimed to fill this gap in knowledge and reported on the decision making process for the selection of essential medicines for SA STG/EMLs and management thereof, over the years, as described by various members of the NEMLC during a qualitative in-depth interview process.

## Methods

### Sample selection

Qualitative studies usually involve smaller samples as compared to quantitative studies as qualitative studies focus on meaning instead of making generalised hypothesis. Large sample sizes are most often unnecessary for qualitative analysis as a single occurrence of a piece of data is sufficient to form part of the analysis framework and one piece of data is as useful as many in creating an understanding of the process behind a topic. A small sample also ensures the researcher can form a more meaningful bond and establish rapport with the participants for a richer discussion and interview process. Furthermore, qualitative research can be labour intensive for very large samples and are sometimes impractical given the time constraints within which to complete the research [9, 10]. Thus, in-depth interviews with 11 participants, who were/are members of the South African National Essential Medicines List Committee, were conducted from January to April 2015. The sample was selected from the full list of members of the NEMLC (104 members) and Technical Expert Review Committees (TERC) (71 members) extracted from each edition of the 10 EMLs published from 1996 to 2012. Participants were then purposively selected based on the number of times they had served on each of the committees as well as the number of levels they served on. This was done to ensure inclusion of all types of members (representatives from all 9 provinces in the country, both previous and new members; members who served on one or both committees, members who served for one or more years; members who served on one or more level of healthcare EML). Invitations to participate in the study were sent to 20 members, of which 11 responded and agreed to participate in the study. Although representation from all 9 provinces in SA was not achieved in the sample, data saturation was achieved with the existing number of participants.

### Development of the instrument

The interview guide for this study was based on the American Society of Health-system Pharmacists formulary questionnaire for formulary management and adapted for the public sector NEMLC in SA [11]. The first draft of the interview guide for the in depth interviews with NEMLC members was piloted with a current member of the NEMLC known to a member of the research team. The interview tool was subsequently refined based on the recommendations made during this pilot interview. The interview tool included open ended questions about committee composition, roles and responsibilities; national policies regarding the committee’s process of selection of medicines and how the committee maintains the list and monitors the rational use of essential medicines.

### Data collection

The interviews were carried out from January to April 2015 and were conducted by the researcher who had no previous knowledge of the workings of the NEMLC and EMLs. There was therefore no prejudgement regarding likely answers to the questions. The researcher was able to limit probing of issues that emerged during the interview thereby eliminating bias and possible influence by the researcher. All except one of the interviews were audio recorded after obtaining consent from the participants. In the case where consent was not granted, detailed written notes were taken.

A review of the NEMLC policy documents available on the NDoH website was also undertaken. This was done to increase understanding of the processes as reported in these documents, to validate responses from the participants during the in-depth interviews and to further verify information obtained from the literature.

### Data processing and analysis

All interviews were conducted in English as all researchers and participants were fluent in the language. The recorded interviews were transcribed verbatim. One recording was inaudible and subsequently excluded from the study. Transcripts were then coded by the first author and verified by the second author. Discussions were held to clarify any discrepancies in coding and to reach consensus. These transcripts and codes were then reconciled and entered into NVIVO, which is qualitative software, to facilitate analysis of the data. A thematic analysis approach was used based on grounded theory where data was coded into hierarchies after an iterative process of classifying codes and re-classifying into sub-codes to ensure no new themes emerged.

### Ethics statement

The study was granted ethical clearance by the University of KwaZulu-Natal Human and Social Sciences Research Ethics Committee (HSS/0154/013). Consent to conduct the interviews with members of the NEMLC was also obtained from the Sector-wide procurement cluster manager at the SA National Department of Health.

For ease of reference and to provide context to the comments from participants, the authors have included the period of service of participants on the NEMLC in brackets after a quote.

## Results

### Response rates and description of sample

Only 11 out of the 20 members (previous and current) originally invited to participate, responded and agreed to participate in the study, namely four from the NDoH including one previously part of the secretariat; five from academic institutions; one from a provincial Pharmacy and Therapeutics Committee (PTC); and one from a provincial Department of Health (DoH). This sample was representative of four of the nine provinces in South Africa namely Gauteng, KwaZulu-Natal, Eastern Cape and Western Cape. These are the four most densely populated provinces (23.7, 19.8, 12.7, 11.2%) [12]. Provinces that were not represented include Free State, Limpopo, Mpumalanga, Northern Cape and North West. Three participants have also Chaired the committees at different times during their term/s of service. The level of involvement of sample participants with the NEMLC and TERC ranges from one term of service for one level to multiple terms of service for one or more levels. Thus, the sample is representative of new and older, longer serving members and is inclusive for all 3 STG/EMLs (PHC, Adult and Paediatric). It is also important to note that there is overlap in service on the committees as some participants have served on both the NEMLC and TERC at different times during their terms. The age range of participants was 37–62 years. Table 1 shows the demographic characteristics of the study sample.

**Table 1:** Demographics of the study sample

Characteristics of sample	
Gender	8- male
	3 - female
Average age	49 years
Age Range	37–62 years
Profession	7 - medical doctors
	4 - pharmacists
Representation on NEMLC	3 - Western Cape
	1 - Gauteng
	3 - KwaZulu-Natal (one from the Provincial PTC)
	1 - Eastern Cape (provincial DoH)
	3 - National Department of Health (1 Secretariat)

The key themes derived from the interviews were: 1. The NEMLC; 2. Medicine selection for the STG/EML; 3. Implementation, monitoring and evaluation of STG/EML; and 4. Change over time. These will be presented separately in the sections to follow.

**1. The NEMLC**

This section encompasses the sub-themes that describe the composition of the committee, how these members are selected, what their roles and responsibilities are, and how the committee develops and manages its policies.

**1.1. Membership, selection of members and conflict of interest declarations**

When asked to describe the membership of the committee most participants were able to describe in general the type of people selected for the committee. One participant responded in detail

“there are content experts, technical experts; there are provincial representatives that are nominated by the head of health or of the province. Each province has a seat around the table. We have representation from the programme within the department of health and of course then we do have, as I said, the normal technical experts. So the chairs of the expert review committee who submits recommendations to the national essential medicines list committee are also part of the EML committee” (Secretariat),

When asked to describe the process of selection of members onto the committee most participants had a very general idea and one participant was unsure. One participant responded:

“Members are selected from pharmacists and medical professionals around the country. Generally, these people would’ve been people active in the provincial PTCs but not always. There’s a wide spectrum of people. Committees are appointed by invitation from the Minister and there is a call for nominations that goes out to most government departments. And it’s upon answering a call for nominations like this that one is invited to participate in the committee. So, there are pharmacists and various medical specialists and public health people and then there are also people from the directorates, and TB directorates, that sort of thing” (2013).

**Fig. 1:**
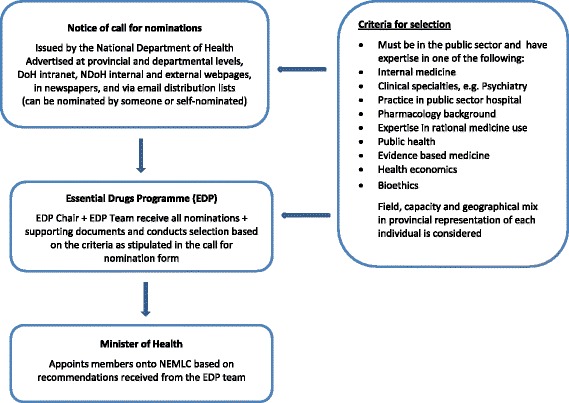
Selection of members for the NEMLC

The following flow diagram (Fig. 1) of the process of selection of members for the NEMLC was developed from the information provided by the participants and a notice of call for nomination form [13].

When the topic of conflict of interest in the membership of the committee was addressed all participants were knowledgeable regarding said criteria. It was apparent from the interviews that a conflict of interest guidance document is made available to committee members at the very first meeting and a declaration must be signed before being appointed. Thereafter a conflict of interest declaration is signed at every meeting to ensure that there is no conflict for that particular meeting as conflict may arise for one meeting but not for another. Responses of participants’ were as follows:

“As an example where now we have a haematologist that’s appointed and he’s a member of the haematology society. So when we discuss blood and blood forming organs chapter, we discuss inhibitors for example, we have to consider his conflict of interest when decisions are made. We might have to, for example, exclude him from discussion if it’s very conflicted or if it’s a minor conflict he can be part of it but not part of the decision making. We have to take it on by a case by case basis” (2012).

“There is a potential conflicts of interest policy or a guidance document which is also available on the department of health website… The chairperson decides how the conflict of interest impacts on the discussions at the meeting. So we rely on members to declare honestly what their conflicts of interests are, we don’t go and investigate their conflicts of interest so it depends on how ethical the members are themselves.” (Secretariat).

**1.2. Roles and responsibilities of the committee members**

The description of the roles and responsibilities of the various members of the committee was compiled from participants’ responses when asked to define their position and role on the NEMLC as follows:

- Chairperson – presides over and conducts the meetings, manage conflict of interest situations and to facilitate discussion.
- Secretariat – forms part of the supporting structures to the NEMLC both administratively and technically. In terms of administrative roles they facilitate the flow of documentation between committees and committee members, facilitate the logistics and travel arrangements for meetings, printing and editing of documentation. They facilitate all communication to and from the committee and the department to external and internal stakeholders. Their technical role is to assist committee members with recording their decisions and to assist with reviews that are undertaken. They also ensure that the committee members abide by the department of health policies and guidelines.

“The secretariats sit on the PTC. Decisions that are made there are minuted. And the idea is that these decisions are fed back to the provinces by the secretariat to the heads of the pharmacy services of the provinces. And the expectation is that those heads then communicate with their local provincial PTCs (PPTCs). And those provincial PTCs speak to the hospital PTCs, regional PTCs” (2013).

“Secretariat is represented at each of these and at national level. And that allows for some smooth flow and continuity and also it keeps the thread going as the members go back to their respective constituencies to do their work before each of the meetings takes place”(1998–2012).

- Technical experts – expert review committee

“To provide technical input as part of the review process, to provide recommendations to the NEMLC”(2012).

- Provincial representatives –

“role is to provide feedback from provincial side of changes that have been made. If ratified, any new items in the province that are non-essential meds and also to provide the review, usually called a one-page review” (2013).

**1.3. The development of NEMLC policies and its management**

Until recently the NEMLC policy documents on membership, conflicts of interest, and terms of reference have not been made available in the public domain. A review of these NEMLC policy documents was undertaken as a triangulation to the in-depth interviews and information found in the literature. There were no reports of in-depth interviews with SA NEMLC members and information on the SA EDP and its processes was limited in the literature. Thus, the cross verification from these different sources helped capture different perspectives of the NEMLC policies and also served to validate this study. The following are summaries of the NEMLC policy documents currently available on the NEMLC page on the NDoH website [14]:

- Declaration of Interests: states that each NEMLC member must review the NEMLC’s guidance document on declaration of interest prior to undertaking this declaration to ensure the selection of medicines for the STG/EML is done in an independent atmosphere which is free of direct or indirect pressures.
- Confidentiality guidance document: This document guides NEMLC members, members of the expert review committees and any working groups established by the committee as to the circumstances in which they should maintain confidentiality regarding the decisions of the committee and its source documents.
- Declaration of Interests Guidance Document: Guides NEMLC members, members of Expert Review Committees and any working groups established by the committee as to the circumstances in which they should declare an interest in the health care industry.
- Terms of reference (February 2013): details the purpose of the NEMLC, their authority to act, composition, conditions of membership, termination of membership, code of conduct, the roles of the Chairperson; secretariat; and various categories of members (technical experts, provincial representatives, major clinical programmes representatives), meeting procedures of the NEMLC, medicine technical report, expert review committees of the NEMLC, general procedure for review of an EML publication, meetings and communications with stakeholders.
- Notice of call for application to the NEMLC (11th October 2016): detailing eligibility criteria for applicants for the position.

An assessment of changes in these policies over the years is difficult to make as these documents, with the exception of the terms of reference and the notice of call for nomination, are not dated. Thus it is difficult to determine when these documents were drawn up, revised or published. Also, there is no repository or archives of previous versions of policy documents if in the event they were revised. The assessment of changes in these policies is therefore extrapolated from the responses of the interview participants.

When asked if the NEMLC consulted other stakeholders to develop its management plans and policies, most participants indicated that there was some consultation although this may not be particularly pertinent to their management policies.

“…We do consult with people. For example, the antimicrobial resistance strategy that was circulated for comment as part of one of the policies we developed to curb antimicrobial resistance and was launched by the Minister after all the stakeholders had been consulted. We do that. Going forward that would be the way we develop policies”. (Secretariat)

“So the cluster management for TB and HIV, they’d be consulted and they’d be shown fresh diseases and sometimes chronic diseases. There should be some sort of collaboration just to keep people informed. I don’t think we’ll ask them to develop policies for us” (2003–2012).

And to the contrary, one member said

“No. Thus far I haven’t seen any consultations happening” (PTC member, 2013).

This was probably due to the short term of service as this was a new member on the committee at the time the interview was conducted.

When addressing the question, When are NEMLC policies reviewed and revised? most participants interpreted this question to be pertinent to the STG/EML itself and responded that it was reviewed on a 2–3 year cycle. Many did not consider the policies surrounding the confidentiality declaration, conflict of interest or the Terms of Reference of the committee in particular and did not comment on these. The responses were varied for those that did provide comment:

One participant was unable to provide a timeframe for review of policies, one said it was “sporadic” (1998–2012) and another said that

“the revision of the Terms of Reference may fall under the entire NEML process and may be lacking” (2003–2012)

Two participants’ responses concurred that NEMLC policies were reviewed on a 3 year cycle upon appointment of a new committee:

“the committee is appointed on a three year cycle so the terms of reference and all the SOP’s and policies are reviewed in the same cycle. And as we progress with the review, if we encounter an issue which requires an amendment to the policy, it is changed in accordance with the changing environment” (1996–2012).

“Okay it’s not very often because it depends on the environment, if there is something in the policy that needs to be reviewed then we do it on an adhoc basis. The national essential medicines list committee policies are reviewed on the appointment of that committee which is after every three years” (Secretariat)

**2. The selection of medicines for the STG/EML**

The process of medicine selection is now encompassed in the review process of the STG/EMLs since the process entails updating the original document produced in 1996. The entire process of decision making for the addition or deletion of medicines on the STG/EML hinges on the following sub-themes: the decision flow system; consultations for decision making; and stipulated criteria for medicine selection. These are described in this section together with other factors influencing the process of medicine selection.

**2.1 Decision making and the decision flow system**

The NEMLC is the decision making body. Their support structures include the 4 subcommittees, the PTCs and the Secretariat. The 4 subcommittees are the technical expert review committees for the PHC, Adult Hospital level, Paediatric Hospital level, and Tertiary/Quaternary level. These subcommittees do the groundwork (literature review, critical appraisal of evidence) for the review of the chapters for the STG/EMLs for those levels and make recommendations to the NEMLC. The subcommittee receives input from the various stakeholders (provincial and institutional PTCs, clinical societies, universities). Representatives from the PTCs are also members of the subcommittees who provide input at ground level from the various hospitals. The secretariat is responsible for the administration and facilitates communication between subcommittees and the national committee. It is also worthwhile to note that the chair of each of the 4 subcommittees also sits on the NEMLC, so these individuals are involved in the review process at subcommittee level as well as in the decision making process at national level. This may be seen as linking the separate committees for easing the decision-making and review processes. Final decisions on medicines selection are made by the NEMLC and presented to the Minister of Health for endorsement. Most participants were aware of the decision flow system between structures and were able to provide detailed accounts of their understanding of the flow of information.

**Fig. 2:**
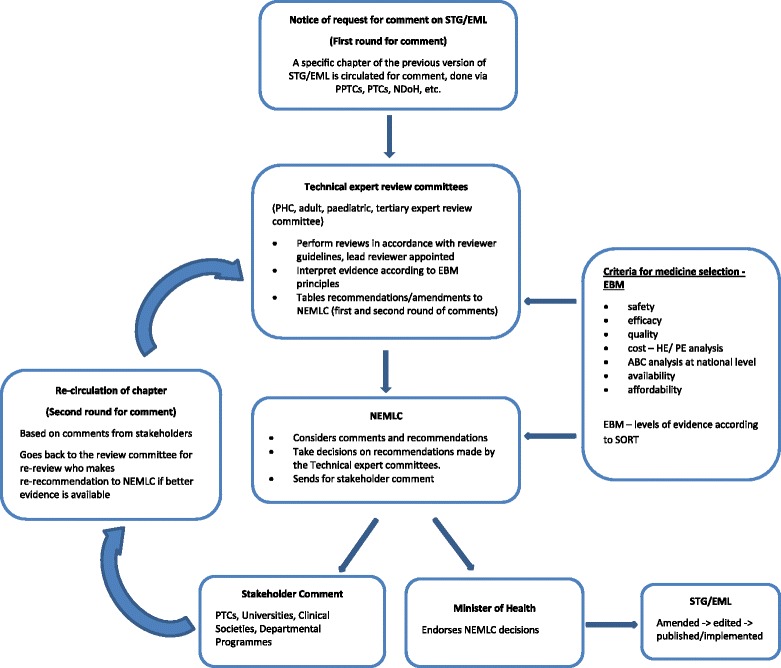
The decision flow system for the decision making process for STG/EML review and the inherent medicine selection process

The following schematic of the decision flow system (Fig. 2) was developed from the information provided by the participants.

**2.2. Consultation for decision making**

When consultation with other committees for the purpose of decision making for medicine selection was discussed, participants responded that consultations may not have happened in the very first committee when the EML was being developed but did occur in later years during the EML review process:

“I don’t recall that we had that mechanism in place at the beginning, it might have been developed later”. (1996)

“The way the constitution of the committee is established is that there is a multi-disciplinary and multi-stakeholder contribution towards the decision making.” (1996–2012).

“there’s wide consultation with all the provinces and with the health science faculties. And we also try and circulate chapters, both draft, both when we starting out with a new process and when we’ve got the final draft we also will include societies, hypertension societies, HIV clinician societies, those kind of societies.” (2003–2012).

**2.3 Criteria for medicine selection and evidence based medicine**

The general consensus from participants of the process of medicine selection, and in particular the process of adding or deleting medicines, is that this process is inherent in the EML review process as represented in the schematic in Fig. 2. This process is followed unless there is a specific reason to reconsider the listing of a medicine such as a medicine safety alert such as detrimental side effects, in which case this is addressed at the next NEMLC meetings which are scheduled regularly. There is a meeting once a week for certain committees. The participants’ responses indicate that the process of medicine selection has always been an evidence based process since the very first EML in 1996. The criteria for selection and use of evidence based medicine over the years have become more robust and stringent. When participants were asked if decisions for medicine selection were evidence based the answer was always “yes”. However, many commented on the limitation being the quality of evidence available to support the decision making process.

“If there’s going to be a change in the guideline, a drug being deleted or a drug being added, then all of the evidence needs to be reviewed”(2003–2012).

One participant provided a detailed account of the process of adding a medicine, which is explained below:

“…well the decisions are made based on, using evidence based medicine principles. Whenever a medicine is added, the question is asked whether or not there’s a medicine that needs to be deleted…they are instructed to look at various things. ABC analysis at a national level, they need to look at tenders, they need to look at pricing, trends internationally, they need to look at departmental guidelines, society guidelines, international guidelines. Also, they are appointed because they have an expertise in a certain area so they should know where the evidence is moving. They then compile the amended changes to the Standard Treatment Guidelines. So, our process is a Standard Treatment Guidelines derived one. If they arrive at a point where they feel that there’s a necessity to add a new medicine then there’s a medicine technical report. That report and the amended Standard Treatment Guidelines are then peer reviewed by the technical subcommittee. The technical subcommittee either sends it back to the reviewer to address or it is finalised in the meeting. Then it goes to National Essentials Medicine List committee. They then approve it for distribution and it is distributed, as I said, to universities, provincial structures, institutional structures as well as clinical societies. Comments are then received within the comment period. Those comments then are reviewed. Hopefully they are supported by evidence. If there is no evidence, we will make a judgement call as to whether or not we will look up the evidence. We will then amend the chapter accordingly. Then it goes to National Essential Medicine List committee and if they endorse it, then it goes for editorial process and that chapter is finalised” (1996–2012).

The most popular reasons provided for deleting medicines were safety concerns, high cost, poor availability; a new medicine becomes available or genericized to replace an existing one.

The principles or criteria for medicine selection as explained by the participants are safety, efficacy, quality, cost-effectiveness, health economic analysis, pharmaco-economic analysis, ABC analysis, Quality Adjusted Life-Years, tenders, therapeutic classes, and cost.

“…we have principles of how we select medicines, we base it on equity, evidence based decision making, we look at the needs, priority of health clinicians, safety, efficacy, equality, we look at affordability and also actual implications of introducing the medicine to the practice environment”(2012).

From the participants’ responses it is evident that the principles of Evidence Based Medicine (EBM) are deeply entrenched in the NEMLC medicine selection process and no decision is ratified unless the evidence has been considered. The NEMLC employs the Strength of Recommendation Taxonomy (SORT) criteria for the grading of evidence in the medical literature and uses the Appraisal of Guidelines for Research and Evaluation II (Agree II Instrument) for assessing and developing standard treatment guidelines from which the EML is extracted.

The SORT taxonomy is a grading scale which allows the application of one taxonomy to many sources of evidence. It speaks to the quality, quantity and consistency of evidence for the assessment of individual studies or bodies of evidence. The framework for this taxonomy focuses on the use of patient-oriented outcomes that measure changes in morbidity or mortality [14].

The AGREE II instrument is used to assess the methodological rigour and transparency in the development of a clinical practice guideline. It provides a framework for the assessment of the quality of existing guidelines; provides a methodological strategy for developing guidelines; and advises on the type of information required and how this should be reported in the guidelines [15].

Members recounted how EBM principles are applied in the EML process as follows:

“So we have a reviewer guideline that gives guidance on how, what kind of information to look at. The committee member who is doing the review is generally the person that will source the information based on the search strategy. You know the whole PICO thing. So we follow evidence based medicine principles. Then we try to impart that to our committee members. Where they are unable to get the articles the secretariat will get it for them. The criteria is evidence based medicines criteria so you have to look at your population and what is the intervention, what is the outcome, what is the comparators etc. Okay, the EML EDP programmes use evidence based medicine principles, even for us as policy makers we use evidence based decision making. Obviously we will look at systematic reviews with high quality randomised control trials as being level 1 evidence, in the absence of that we look at large studies with a properly conducted randomised control trial. Then our quality of the evidence decreases as we go along. If we don’t have any systematic reviews of good quality trials, then we will look at the single trial, you know single randomised control trial. In the absence of that, then only we look down the chain for other evidence. So then we would look at observational studies or case control studies in the absence of a randomised control trial or a systematic review. Clinical trials are evaluated and critiqued so we do that also as part of the reviewer handbook” (Secretariat)

**2.4 Other factors affecting the selection of medicines**

A number of other factors impacting on the decision making process for medicine selection emerged from the interviews. These include the roles of: (a) information from pharmaceutical manufacturers, (b) therapeutic classes of medicines, (c) tenders and cost considerations. These are discussed below.

When asked about the use of information provided by manufacturers in the selection of medicines, all participants agreed that information from pharmaceutical companies is not influential in decision making. Some have indicated that although this information may filter through to the committee via a member it is not used in the decision making. Dossiers from pharmaceutical companies are not considered and are viewed as bias. Participants’ responses include:

“…in my experience the committee views published pharma-economic analyses, especially with heavy influence from the manufacturers, with a great degree of scepticism” (2013).

“We’ve had a few comments from outside experts where we strongly suspect that the pharmaceutical companies are behind them but we don’t use that dossier. We look for our own evidence, so we never use the manufacturer’s dossier because it’s never formally presented to the committee” (2003–2012).

On careful examination of the STG/EMLs over the years, one will note the listing of therapeutic classes of medicines instead of the listing of a particular medicine as a treatment. This is related to the tendering and procurement processes. Sometimes, this tendering process impacts on the medicine selection process and medicines may be selected because they fall within a particular therapeutic class as explained by one participant:

“they may say a therapeutic class, so they will say for macrolides, let’s go with Azithromycin, Clarithromycin, …then they’ll say let’s advertise it as a therapeutic class for the sake of the tenders. So if the tender comes in cheaper for one macrolide over the other, one will be chosen and the other will be removed. So that’s why they are now citing examples in the EML” (2013).

“….so we compare prices on the tender price, so then we can select the most affordable medicines” (20013–2012).

Health economic considerations also play an important role and participants have indicated that cost is not the driving factor in the medicine selection process. Many have indicated that some kind of pharmaco-economic analysis is performed during the selection process but the general consensus is that it is currently not a very comprehensive process and could be improved upon.

“….it’s not just the cost of the medicines but we try and express costs in terms of changes in quality adjusted life years. It’s not always that easy but we do a rough estimation we don’t do a formal health economic analysis, it would just take forever if we did that. But we are very mindful of costs and we are not taking the attitude that if it’s expensive we can’t have it. We look very carefully at the efficacy of the medicine. And then also there are different people within the Department of Health whose job it is to negotiate with companies. So if we feel that a particularly costly item is important, it’s kind of their function to negotiate a better price and not the NEMLC function. But clearly in a public health system like ours, cost is a big factor” (2003–2012).

**3. Implementation, monitoring and evaluation of EML policy**

The monitoring and evaluation (M&E) component of an EML policy is vital for the successful functioning of an EDP. This aspect provides information to policy makers on how the policy and documents are being received and accepted by those who need to implement it. It further provides valuable information on which parts of a policy are working and which are not, critical for policy review and reform. The results of this study show that this component is severely lacking in SA STG/EML programme. Participants were asked a few questions to ascertain the degree to which M&E took place, viz. Are dates established to reassess the effect of an STG/EML decision on the quality or cost of care? And how much later after inclusion of a medicine on the STG/EML is this assessed? Are outcomes monitored? Are there any reports on monitoring the use of medicines by patients?

Responses suggested that no formal assessment was done to evaluate decisions taken, no patient outcomes were monitored by the NEMLC but rather they depended on information from the PTCs who were responsible for this type of monitoring, and monitoring of medication use.

“There’s no real assessment on the effect of a decision on the quality or cost of care… Not a formal process” (2003–2012).

“So the monitoring and evaluation of STG/EML is problematic. DURs (Drug Utilization Reviews), that’s a major problem, that’s a flaw. So we do ABC analyses and all that, but there’s a flaw, we don’t really know if it’s been used properly and what the DUR has done. So it’s a major issue, DUEs (Drug Utilization Evaluations) is not done at this moment in time. And that certainly needs attention” (2003–2012).

Other challenges or shortfalls affecting the STG/EML implementation, monitoring and evaluation processes identified by participants include: (a) communication processes between structures (national to provincial and departmental) and the dissemination of information along these lines; (b) the lack of health economic expertise in the country; (c) the lack of country specific health economic information impacting on health economic analyses; (d) poor outcomes monitoring and evaluation of STG/EML decisions and rational use of medicines by patients; (e) blurred lines of roles and responsibilities between committees (PTCs, PPTCs, NEMLC); (f) alignment of processes (medicines selection with procurement) in the STG/EML process; and (g) challenges with availability of appropriate paediatric medicines. Table 2 describes the opinions of participants in relation to these challenges.

**Table 2:** Participants’ opinions on challenges/shortfalls in the NEMLC and their processes

Issue	Comment
Communication processes	“I think one of the weaknesses in the committee is communication. They do communicate to the provincial people but the provincial people then don’t always circulate it around. It is sent to all health sciences faculties but then maybe it just dies in the Dean’s office and doesn’t get sent around. When people give comment, feedback is seldom given to the individual commentators. Actually you know you can’t expect the secretariat to do everything. But the committees recognise that communication back to people on the ground is an issue. Our main message of communication in a way is the book, especially the primary care book. There’s a road show to provinces where new changes are highlighted and explained and then the book is launched. There’s a team from DoH that goes down to each province to explain that. So they try hard but it’s always hard to get down to the hundreds of thousands of health care workers on the ground but that’s the process”. (2003–2012) “The way of disseminating information is cumbersome and essentially goes to depots and pharmacists rather than directly targeting prescribers, which is a problem”. (1998–2012) “This has been one of the biggest challenges, I think. The movement of information from the EML committee to the end users is something we’re working hard on. It’s an extremely difficult task because there’s so many role players involved. There have been problems and complaints from provinces that the decisions are taken and no insight into why certain decisions are being made. There’s very little opportunity for feedback and when there’s feedback given there’s very little feedback on the feedback. So, the committee have taken up publishing rationales for every decision. Those are available on the website, and can be circulated to people with queries, explaining what the rationale was for a particular decision, reviewing the evidence base, doing the cost analysis, etc.” (2013) “The communications between clinical advisors and pharmacist is robust”. (1998–2012) (exception)
Health economic expertise	“So there is a lack of health economic expertise in the country. And within the committees there are people with extra training in health economics and with good insight into it. But we do not do formal health economic analyses on anything. We don’t have the time or the personnel to do that. But as I said earlier, we don’t just look at cost; we try and express cost per quality adjusted life year. We use the rough world bank, WHO figure. That one to three times per capita GDP per QALY is something to be considered. More than three is too much, under one is good. But it’s not formal. It’s more a rough estimation to see whether we’re in the ball park rather than a formal process. It would be great if we had the resources of NICE but we don’t. But we do aspire to it. So there are only a handful of people with skill in the country. The EML keeps them all busy all the time and the EML has no budget for this. So we see it as crucial but lack the expertise”. (2003–2012)
Lack of Health economic information	“All of those were considered if the information was available. So anyone of those, whatever information, because there often isn’t information on this particular subject although it’s considered important, in my latter days it’s considered even more important as these disciplines were becoming more evolved. But there was always insufficient information, but all of the criteria that you considered; they are all methods that were used if the information was available”. (1998–2012) “The answer is no because first of all there are many pricing considerations in the public sector which are not relevant to international environments. So international cost effectiveness, or shall we say pharmaco-economic studies are very rarely directly applicable to the South African environment. Again in the South African literature there have not been many publications of pharmaco-economic evaluations as they pertain to the public sector”. (1996–2012) “So one of the problems is that these types of studies are very rarely available. The pricing committee put out guidelines for conducting pharmaco-economic studies. So the government have tried to facilitate the publication thereof. So although I said no we don’t use them, we would love to use pharmaco-economic evaluations that are appropriately targeted to the South African environment if they were readily available to us. We do look at international studies but usually the international pharmaco-economic evaluations we use as a basis for constructing our own, so that we don’t have to reinvent the wheel but we rarely just take one without adapting it to the South African environment”. (1996–2012)
Outcomes monitoring and evaluations	“So I think what I would say is, we aspire to that however the capacity in the country at the moment does not support that but basically we have fantastic pharmaco-vigilance policies, as you know KZN has won partner, spent millions of Rands on pharmaco-economic incentives and those sort of things” (1996–2012). “At the moment people will give you answers as we think about it, but the reality is that the fantasy is not met. And also remember at the national level we would be reliant on provincial competencies then and that’s going to be very difficult. And one of the things that I lament all the time is, if you are sitting in graduation at the universities and you look into all the funny thesis that people do, you wonder whether or not they’ll fit in South Africa because there’s very little, ever, in academia or in our provinces where anyone ever looks at outcomes. So basically we are very reliant on international publications. Obviously if you get a local publication we get very excited but they are very very rare”. (1996–2012) “The only internal data that we have readily access to, again because we haven’t been collecting it, because it’s not a South African tradition to do so, is the information on the drug utilization, essentially. We basically have dispensing data, which is becoming more detailed and of higher quality but that really addresses issues of affordability more than anything else. And some questions of equity when you find a medicine is being used in one area and not being used in another”. (1998–2012) “The only mechanisms I recall at the time was to ensure that all prescribers had copies of the EDL and although this was not done within government itself you had NGO’s running courses in drug supply management including prescribing and dispensing. I think a lot more could have been done internally. I think this was a weak point on the part of the government at the time we established the EDL”. (1996) “So the monitoring and evaluation of EMLs is problematic. DURs, that’s a major problem, that’s a flaw. So we do ABC analyses and all that, but there’s a flaw, we don’t really know if it’s been used properly and what the DUR has done. So it’s a major issue. DURs is not done at this moment in time. And that certainly needs attention”. (2003–2012) “It is a process I have criticised internally and externally as being short on review of its impact and whinge on process, they keep on doing the same thing again and again but I think people are starting to accept that we may need to look a bit harder at impact rather than just development”. (1998–2012)
Roles and responsibilities of Committees	“I think there’s quite often confusion between the delineation of the National Essentials Medicine List committee and that of pharmaceutical services….generally there’s confusion about the role and responsibilities of the two”. (1996–2012)
Aligning processes – selection and procurement	“It became clear that there has not been much interaction between the process of selection and that of procurement. Quite often they will decide to select a medicine based on efficacy and even cost without looking at how widely available it is. We have dozens of patients on treatment, failing the course, yet the medicines are supposed to be good but it’s not readily available. And when you start putting patients on the medicine, then you have shortages and patients lapse and so on. I would say that even up to a few years ago, there has not been a strong link between selection and procurement”. (1996) “I don’t know if it’s called a policy but they do have a SOP in place now to address medicine product shortages. They have a formulary against which you check to see if the product is on tender etc. but the whole process is unclear. It’s not like a policy; it was just like a circular that was sent around. There is a problem at present with transition in tender and there are currently huge medicine shortages”. (PTC, 2013)
Challenges with paediatrics	“One thing is, being a paediatrician and knowing that it’s a particularly difficult issue with paediatric drugs passed… they are a particularly vulnerable group of patients. My personal opinion is that paediatrics needs its own Standard Treatment Guideline process because it’s very different. Children should be prioritised not adults, according to the constitution. So I think those two are important aspects. I think the committee focuses on a lot of adult guidelines that isn’t the same and demands huge evidence burdens which isn’t available for children and which might result in lack of availability for children”. (2013)

Further to the questions of our interview guide, one of the participants posed extremely valuable questions which addressed the future of the EMP. This was a step further than the aims and scope of this study, but is well worth mentioning as it is vital to the EMP and STG/EML process in its entirety. The authors strongly recommend and will investigate these questions as future studies as it impacts greatly on the strengthening and sustainability of the overall STG/EML process. The participant’s concerns are as follows:

“…is the process itself cost effective, or is there funding around it?”

“Is there not a review committee there itself,… why are consultants, typically experts, doing the review?”

“You may want to know why manufacturers don’t give dossiers… why we don’t use the medical aid dossiers, or why we don’t use data which has been looked at already by, for example, the funding industry and the private funding industry”

“…what is the succession planning, is there training, do you train people to review?”

These are extremely important questions that if adequately addressed could have huge consequences on the cost of running the EMP and its overall impact in terms of (a) developing guidelines for the use of information from the private sector as well as some credible information from the manufacturers; (b) improving human resources for the STG/EML review process in terms of providing adequate training on the EBM principles and the review process itself and (c) ensuring sustainability of the EMP by improving succession planning initiatives in terms of providing opportunities for capacity development of health professionals for these roles.

Some recommendations made by the Lancet Commissions (2016) [16] on making medicines more affordable by way of using Health Technology Assessment may prove useful for the monitoring and evaluation component of the EML policy and decision making process for medicine selection in SA. The Health Technology Assessment method assesses the value of a new medicine. It is a multidisciplinary mechanism that examines the safety, clinical efficacy, effectiveness, cost, cost effectiveness, organisational implications, social impacts, ethical and legal consequences of the application of a drug or other health technology. Health Technology Assessment is a method that generates evidence to support procurement and reimbursement for essential medicines selection and can be used in price negotiations. Middle income countries that have implemented Health Technology Assessment include Poland, Malaysia, and Colombia. SA can learn from these countries experiences and adapt to our setting. This would prevent double work, especially for new expensive medicines.

**4. Change over time**

From the interview data an overarching theme describing the change over time emerged. This is described in Table 3. which illustrates the progress made over the years for both the NEMLC and its processes in terms of (a) the improvement in time to review the STG/EMLs; (b) more stringent criteria for medicines selection; and (c) development of committee policies and processes to more clearly address and define membership, conflicts of interest, roles and responsibilities.

**Table 3:** Participants’ views on how the NEMLC and its processes have changed over time

Issue	Comment
Time period for revision of EMLs	“So standard treatment guidelines, the last one was published in 2006, for paediatrics for instance, and it’s just been reviewed. And there’s a recognition that this time period was too long and that it should be shorter and so we hope to be able to review it in a much shorter time frame in the future. So I think the plan, we’ve already started to, just after the publication of this one; we started the review of the next one. So the idea is almost yearly or two-yearly and especially once we move on to an electronic database that the standard treatment guidelines can be updated almost continuously as things change, evolve”. (2013) “I would say at this point yearly probably now. With every review of the STG we do it yearly. Prior to this it was happening every few years you know since 2006. That was more like eight years, but the idea is to increase the turnaround at the time” (2013)
Changes in selection criteria	“Prior to, I’d say 2005, we looked predominantly at acquisition price but since 2005 there has been a systematic inclusion of pharma-economic principles. And currently no medicine is added without at least a consideration of the pharma-economic”. (1996–2012) “I seem to recall at that time even though we were more interested in efficacy regardless of the cost therefore we rarely looking at cost effectiveness analysis. There was a pressure also to consider the cost of a drug, because we wanted to lend selection to procurement. Although for a long time that hasn’t happened. So we didn’t really do cost minimization for the time, any kind of consideration at the time, I think things improved over time, for the first few years, I wouldn’t say we were that strong in our evaluation of the cost effectiveness of medicines”. (1996)
Committee policies and processes	“Potential conflict of interest in the membership of this committee, we probably didn’t have much in place at the beginning, but with time I think with experience we realised that something needed to be developed that would be signed by members, something that can ensure that at the beginning of the year they would declare anything that would bring them into conflict, but I cannot vouch that we did it from the second year, it might have been years before that actually happened”. (1996) “Are other committees consulted? Yes, of late now with the advent of pharmacists that are PTC committees. I think that is happening”. (PTC, 2003) I don’t know if it’s called a policy but they do have a SOP in place now to address medicine product shortages. They have a formulary against which you check to see if the product is on tender etc. but the whole process is unclear”. (PTC, 2003) How often are committee policies reviewed? “I don’t recall that we had that mechanism in place at the beginning; it might have been developed later”. (1996)
General comments	“The only comment is that this has been an evolving process, we’ve learnt through our own educational process, trial, error and trying to explore. And that process has been evolving and continues to evolve. But in effect I think it’s been highly successful and certainly from where we started off with nothing, I think we were able to produce something that was exceedingly successful and we very grateful that we were able to do that work”. (2003–2012) “The South African one was developed from nowhere and actually has come quite a long way but I think it is time for it to be revisited and there are attempts to look at where we are going in the future and perhaps to re-evaluate what we are doing and why we are doing it, clearly a blessing to a guideline committee and more into a strict formulary thing which would be easier to measure”. (2003–2012) “Well for us, I mean, although we had information from others who had experience in their own right, and there have been attempts previously, by previous health departments to have some kind of formularies or lists, this was really a new experience and I think it took time before we managed to establish ourselves as department of health and EDL committees that were sort of well-established and was able to follow international best practices. I think it took some time before we could get to that, you know, we started by crawling, gradually over time I think things would have become much better. I recall that we also used to comment at the WHO, we would advise on one or the other mechanism of doing reviews. It was really quite an experience, new experience for us”. (1996) “It is a very thorough process, people that are not on NEMLC would think who are these people sitting and making decisions but it’s a very thorough process and it’s a very transparent process”. (2013)

## Discussion

In summary, the results from the interviews show that the NEMLC has undergone tremendous transformation over the years. Although the membership of the committee largely remains unchanged, the committee has developed and improved its policies and processes over the years. The selection of NEMLC members, their roles and responsibilities are now more clearly defined in policy documents. Conflicts of interest are dealt with in a formal manner with mandatory declarations by members upon appointment and at each meeting. These documents have also recently been made available on the NDoH website. This makes the processes more transparent as the information is now available in the public domain which was not the case previously.

The process of medicine selection has transformed into an extremely rigorous evidence based process. The process of medicine selection and process of review of the STG/EML are underpinned by evidence based decision making. The time to review STG/EMLs has improved over the years and the process is now almost a continuous one with reviews of chapters occurring as an ongoing process.

However, much attention and improvement is required for the monitoring and evaluation component of the EML process. There are also recommendations to address the issues of sustainability of the NEMLC in terms of its succession planning, capacity building, and mentoring and training in EBM principles and review of the STG/EML.

### Main findings

The WHO has a Model list of essential medicines which serves as a guide for countries for the development of their individual country specific EMLs. It is recommended by the WHO that the process of medicines selection be based on safety, efficacy, quality, and comparative cost-effectiveness [17]. Other influencing factors include pharmaco-economic and health economic assessments, Quality of Adjusted Life-Years, cost, budget, tenders, price negotiations, therapeutic classes all affecting medicine selection. National EMLs should be used to guide procurement, supply and use of medicines in the country. Currently there are over 156 countries who have adopted an EML including SA.

The SA NEMLC is a multidisciplinary, multi-stakeholder committee with the secretariat and members from the PTCs as supporting structures. The participants have stressed the independence and autonomy of the committee in the medicines selection process although other guidelines from other local and global organizations and committees such as those made available for HIV, TB, and Asthma for example, may be considered but do not completely influence the decision making process. Participants have described the process as “transparent” and “evidence based”. The concept of EBM is rooted in the medicines selection process and no decision is made without first considering the best available evidence. This is evidence of one of the many changes over the years as described in Table 2, which is a synopsis of notable quotes from participants which indicate how the NEMLC processes have changed over time.

### Comparisons with other studies

The WHO concept of essential medicines has gained global acceptance. Currently 4 out of 5 countries worldwide have a national EML, making the WHO Model EML a cornerstone in essential medicines policies [17]. SA is one of these countries to have effectively adopted and developed a national EML since 1996, based on WHO guidelines. The overall structure of the SA STG/EMLs has remained largely unchanged over the years. This is consistent with the WHO Model EMLs which have also maintained their structure but different in that the WHO Model EML medicine selection process has matured and has moved from a more experience-based approach (used before 1999) to a more evidence based approach (adopted since 2002) [5] whilst the SA EMLs medicine selection process has always been evidence based with the process itself becoming more stringent over the years. Post 2002, the WHO expert committee refined the medicine selection process to include evidence for public health relevance, efficacy, and cost-effectiveness. In addition, guidelines also now include input from various stakeholders who sit on the expert committee. These criteria are all similar with the current process in SA [18].

The process of EML review for SA and the WHO is very much the same and this is discussed below. Both processes apply evidence based approaches for medicine selection using a number of criteria viz. the disease burden, availability of sound and adequate evidence on safety, efficacy, and comparative cost-effectiveness of available treatments, although the pharmacoeconomic aspect still requires some strengthening in SA. Furthermore, where availability of evidence is inadequate The WHO Expert committee either waits for more evidence or bases its decision on expert opinion or experience. The same is true for SA, where value judgements are made and expert opinions or lower levels of evidence are considered, such as case reports or consensus opinions. The process of applications for amendments, additions, deletions of medicines to the WHO Model EML is submitted via relevant departments to the secretary of the WHO Expert committee. In SA, this process occurs through the EDP who receives all motivations for amendments to the EML. The selection of members onto the WHO Expert committee is done by the Director General from the WHO Expert Advisory Panels and members are selected based on gender balance, geographical representation and professional competencies. All potential conflicts of interest are declared by the members and captured in the respective Technical report series. The member selection is similar in SA with the exception of gender balance and the NEMLC is appointed by the National Minister of Health and there is also a conflict of interest declaration made by the members of the NEMLC.

The WHO process differs in that all applications for amendments must be received four months prior to the meeting of the Expert committee and all application reviews/comments are made available on the webpage and verified for completeness by the secretary of the Expert committee. The final outcome is summarized and published in a WHO Technical Series Report. This process is very transparent and SA has not adopted this latter phase of the medicine selection and review process as yet. The WHO process also allows for patient advocacy groups to comment on the various applications and draft recommendations although these are not used in the decision making part of the process. This is also not something currently considered in SA.

Australia has a Pharmaceutical Benefits Scheme (PBS) which pays for most prescription medicines. It is one of many government programs which applies evidence-based decision making to the funding of health technologies. PBS processes support the provision of affordable, equitable access to prescription medicines and is not a cost containment tool. Similar to the NEMLC in SA, Australia has The Pharmaceutical Benefits Advisory Committee (PBAC) which is an independent statutory committee, appointed by the Minister of Health, and is responsible for the selection of medicines onto the national formulary. PBAC are advisory to the Minister for Health and Ageing who takes the final decision to list a medicine. The PBAC process of medicine selection is also evidence based as is the case in SA. They also take into account both comparative effectiveness and comparative cost-effectiveness of medicines during selection [19].

Botswana developed its National drug policy in 1999. The first Botswana EML was published in 2005 and revised in 2012. The lag in revision is similar to the first set of SA EMLs. The committee responsible for medicine selection is the National Standing Committee on Drugs. The Botswana EML was adapted from the WHO Model EML (April 2003 version). Criteria for medicine selection was based on WHO guidelines. The National Standing Committee on Drugs engaged in a consultation process with Drugs and Therapeutics committees at different levels of health facilities during development of the EML whereby a draft of the EML was circulated for comment [20]. The central medical stores is responsible for procurement of medicines and medical supplies for the public sector. There is also a monitoring component of the EML process whereby the central medical stores collects usage and consumption data on quantities of medicines received and distributed and the cost of these medicines via a computerized system [21].

From the above discussion on some country specific EML processes it is evident that SA’s EML process aligns well with the WHO process.

The successful implementation of a medicines programme is the evaluation of the medicines policy and continuous monitoring of the use of medicines. Knowledge of the impact of the policy is vital for further development [22]. Some national medicines policies may address the importance of M&E, but the actual indicators for monitoring or the framework for monitoring are often lacking within the policy itself [23]. The challenges experienced by NEMLC members in the SA EML programme in terms of communication between departments and committees, the lack of technical expertise for health economic analyses, the difficulty with alignment of processes, and the poor M&E of policy is not unique to SA. These are common constraints encountered by countries employing any national medicines policy, as explained in a policy and legal framework document for National medicines policies published by Management Sciences for Health (2012) [24]. A list of indicators to assist with monitoring medicines policy has been developed by the WHO and may be adapted to suit the needs of specific countries as every country’s medicines policy is unique to their situation and need [25].

It is explained that there are many actors involved in the efficient running of a medicines policy, from the national government departments of health, trade and industry, and finance, to health professionals, academics, public and private wholesalers, retailers and local and international pharmaceutical manufacturers, provincial and district health departments, and NGOs. This diverse group of participants makes the efficient running of a medicines programme multi-faceted and difficult. An efficient system to monitor and evaluate a medicines policy must be in place as it serves as a management tool for the assessment of progress of the policy. It assists policy makers to make appropriate management decisions to respond to identified problems with the policy and engage in discussions with national stakeholders to problem-solve and improve the performance of the programme. A strong partnership from all actors involved is required for a successful medicines policy process [24].

A study conducted by Roughead et al., 2016 [22] describes a few countries experiences with monitoring systems for the use of medicines and may serve as examples that SA can use to strengthen its M&E aspect of the medicines policy. These are summarized below.

Bhutan has an essential medicines and technology division which provides systematic, evidence based information to the Ministry of Health. Progress of the policy is evaluated using a subset of the WHO indicators for monitoring medicine policy together with DURs. Monitoring involves analysing if medicines belonged to the NEML and the number of medicines on each prescription.

Indonesia has a rational use of medicines programme that monitors medicine use at all health facilities in the country. Data is collected, using the WHO indicators, daily at health centres, monthly at district or municipal offices and sent to the minister biannually for compilation of the annual national performance indicators report

Malaysia monitors medicine use by using numerous data sources viz. National Pharmacy Service Statistics, National Medicines use survey and the National medicines price survey. The data is used to assess utilization, prescribing patterns, quality of healthcare, pharmaceutical expenditure, price variations and to monitor and evaluate the effects of interventions for rational drug use.

The Republic of Korea does not have a formal national medicines policy but has a system of National health insurance. A national e-health platform allows data collection for medicine monitoring purposes. The country also has a DUR programme.

### Strengths and limitations

We gathered perspectives and opinions from past and current members of the NEMLC about their experiences with the committee, its procedures and processes surrounding essential medicines selection over the years. The sample was representative of the wide spectrum of members on the committee as members from the NDoH, provincial DoH, and PTCs participated. In addition, we were able to recruit NEMLC members from every committee for every level of the NEMLC from the very first EML in 1996 to the 2013 committee. The sample was not geographically representative of all 9 provinces, although the 4 largest provinces were represented, and valuable opinions from these individuals have not been captured in the study. The element of recall bias must be considered for all participants, especially for those participants who were from the very first NEMLCs from 20 years ago. Furthermore, the familiarity with NEMLC processes may have been over-reported as participants were provided the opportunity to peruse the interview questions prior to the interview.

### What this study adds and suggestions for future studies

This research has highlighted the accomplishments of the NEMLC and the EMP, over the years. The research has also brought to the fore, the poor monitoring and evaluation component of the current EML system. Although this is listed as a requirement of the NDP for SA, 20 years later it still remains fairly unfulfilled. The authors recommend that this component of the EML policy be strengthened as it is one of the critical building blocks for an efficient, informed, sustainable EML process and impacts largely on attaining better healthcare for all citizens. Further studies are required to investigate the actual reporting mechanisms and reporting committee functions from the ground up at a local and PPTC level that feeds and supports the M&E component, headed by the NEMLC. Studies probing the total financial consequences of the listing decisions such as budget impact analysis of adding medicines must be explored and clarity on how the committee establishes a medicine to be “affordable” must also be further investigated. These studies will contribute to a better understanding of how decisions are made taking the country’s budget into account. SA can capitalize on the experiences from other countries such as Australia, Norway, Canada and Italy where budget impact assessment is a formal requirement in the reimbursement decision making process [16].

It is important to note that the development of an EML is only the starting point of the EDP process; it must be effectively linked to the processes for procurement, supply, training, and monitoring and evaluation of prescribing and medicine use for it to make a positive impact and valuable contribution to better healthcare.

From the discussion above it is evident that indicators or some kind of performance standard is required to assess the progress of a policy or to assess the effects of policy decisions that result in changes in the policy. For this process to be effective major resources including human resources for data collection and interpretation are required. Furthermore, budget impact analysis of committee decisions on medicines selection is lacking and needs to be expanded upon.

## Conclusions

This is the first study in SA to report on how decisions are taken to include or exclude medicines on SA STG/EMLs and provides insight into the SA STG/EML medicine selection, review and monitoring processes over time.

There may be opportunities for the SA medicines policy to be strengthened by taking a leaf from other countries’ experiences as described above. Further studies are required to confirm this. However, the lessons learnt from the SA experience with STG/EMLs and medicine selection over the last 20 years serves as a noteworthy example for other low-middle income countries when developing their own NEMLs or even for the process of STG/EML review.

## Abbreviations

DoH: Department of health
EBM: Evidence based medicine
EDP: Essential drugs programme
EML: Essential medicines list
M&E: Monitoring and evaluation
NDoH: National department of health
NDP: National drug policy for South Africa
NEML: National essential medicines list
NEMLC: National essential medicines list committee
PHC: Primary health care
PPTC: Provincial pharmacy and therapeutics committee
PTC: Pharmacy and therapeutics committee
SA: South Africa
STG: Standard treatment guidelines
TERC: Technical expert review Committee
WHO: World health organization
